# Increasing consumer engagement: tools to engage service users in quality improvement or implementation efforts

**DOI:** 10.3389/frhs.2023.1124290

**Published:** 2023-07-25

**Authors:** Eva N. Woodward, Irenia A. Ball, Cathleen Willging, Rajinder Sonia Singh, Celia Scanlon, Damon Cluck, Karen L. Drummond, Sara J. Landes, Leslie R. M. Hausmann, JoAnn E. Kirchner

**Affiliations:** ^1^ Department of Psychiatry, University of Arkansas for Medical Sciences, Little Rock, AR, United States; ^2^ VA Center for Mental Healthcare & Outcomes Research, Central Arkansas Veterans Healthcare System, North Little Rock, AR, United States; ^3^ Pacific Institute for Research and Evaluation, Southwest Center, Albuquerque, NM, United States; ^4^ Arkansas National Guard Foundation, North Little Rock, AR, United States; ^5^ Behavioral Health Quality Enhancement Research Initiative (QUERI), Central Arkansas Veterans Healthcare System, North Little Rock, AR, United States; ^6^ South Central Mental Illness Research Education and Clinical Center (MIRECC), Central Arkansas Veterans Healthcare System, North Little Rock, AR, United States; ^7^ VA Pittsburgh Healthcare System, Center for Health Equity Research and Promotion, Pittsburgh, PA, United States; ^8^ Division of General Internal Medicine, Department of Medicine, University of Pittsburgh School of Medicine, Pittsburgh, PA, United States

**Keywords:** service users, consumer, patient engagement, patient and public involvement, community engagement, implementation science, quality improvement

## Abstract

**Introduction:**

Engaging service users or consumers in quality improvement or implementing a new service is important across settings and may reduce health inequities. Implementation strategies leveraging consumer engagement are neither commonly used nor robustly operationalized in implementation science. Implementers (e.g., middle managers, facilitators) want to involve consumers in implementation activities, but do not always feel confident in how to proceed. We developed a compendium of tools called Consumer Voice to guide others how to engage consumers in design/delivery of implementation strategies. Although generalizable to other settings, we developed Consumer Voice within the context of implementing suicide prevention treatments in healthcare to reach rural U.S. military veterans, as there are suicide inequities for people in rural areas.

**Methods:**

We developed Consumer Voice using a multistep process and human-centered design methods. In between steps, a design team met to generate insights from data, and decide which prototypes to create/refine. In preliminary work, we conducted a scan of examples in healthcare of patient engagement in implementation activities and interviewed two implementation experts about preferred learning styles. In Step 1, we interviewed 26 participants with experience in community engagement, implementation, or lived experience as a rural U.S. veteran with suicidal thoughts/behavior. In Step 2, 11 implementers beta tested prototypes then share feedback in focus groups. In Step 3, we reconvened participants from prior steps to review tools and, using nominal group technique, prioritized remaining recommendations.

**Results:**

Consumer Voice is online, modular, and nonlinear for self-guided learning tailored to beginner, intermediate, or advanced experience with consumer engagement. Tools consist of slides, audiovisual content with written text, and templates. Findings indicated there is not one “right” way to engage consumers in implementation activities, rather that implementers wanted tools showcasing core principles for consumer engagement and practical ideas.

**Discussion:**

Consumer Voice can be used by implementers to reflect and decide on how to apply consumer engagement implementation strategies to improve equitable dissemination and uptake of evidence-based practices. Most insights generated by user data were explicitly to build trust between consumers and professionals representing institutions, which may be one component to reducing healthcare inequities.

## 1. Introduction

Engaging consumers of innovations (i.e., service users, end users) to facilitate equitable demand for and uptake of innovations is important across a wide range of settings (1–3). Consumers are the people who use, receive, or are most affected by an innovation, which could include new policies, treatments, or programs. Examples of consumers are patients in healthcare settings, students and families in education settings, and incarcerated individuals in criminal justice settings. When implementing an innovation in new settings, implementers—quality improvement personnel, implementation scientists, and practitioners—typically focus on changing dynamics within an organization, the processes within smaller units of local context, and the behavior of people delivering an innovation (4). “Consumer engagement implementation strategies,” defined as those that focus on people who are direct recipients of innovations and practice changes, are less commonly used (5) and not robustly operationalized in the implementation science literature (4, 6).

Experts identified five consumer engagement implementation strategies to enhance uptake of innovations. They include (a) involving consumers or family members in implementation or quality improvement activities; (b) intervening with consumers to enhance their own uptake of and adherence to an innovation; (c) preparing consumers to be more active participants in their own services; (d) increasing consumer demand for innovations; and (e) using mass media to disseminate information about innovations (4). Unpacking the first type—involving consumers in implementation or quality improvement activities—might include having consumers serve on advisory councils (7), be practice change agents who assist with innovation implementation (8), marketing, or dissemination (9); and/or participate in user testing of consumer-facing products (10). What these strategies have in common is their direct involvement of consumers to inform and/or participate in the implementation strategies used to spread uptake of an innovation. Although implementers may want to involve consumers in implementation activities, implementers do not always feel confident in how to do so. Despite increasing requirements by payers and organizations to engage consumers in implementation or quality improvement (1, 11–13), using consumer engagement implementation strategies, alone or in conjunction with strategies targeting deliverers of innovations and their organizations, appears to be uncommon (14, 15). When engaging consumers in implementation activities, implementers face numerous challenges, such as uncertainty about usefulness of engaging consumers, confusion about terminology, lack of role clarity, or lack of funding to do so (1, 16, 17).

To increase the use of consumer engagement implementation strategies and specifically to clarify *how* to involve consumers in implementation or quality improvement activities, we engaged in a multi-step, systematic process to develop a compendium of tools called Consumer Voice. Designed to support implementers, Consumer Voice was developed within the context of implementing a suicide prevention intervention—Safety Planning Intervention—in rural primary care settings to reach rural U.S. military veterans in Arkansas, as suicide rates are double among rural-dwelling (vs. urban) veterans (18, 19). However, Consumer Voice tools were designed with generalizability in mind and can support uptake of any innovation in any setting. We believe using Consumer Voice would likely result in either (a) greater use of other types of consumer engagement implementation strategies by implementers (e.g., increasing demand for innovations) or (b) consumers assisting or leading the design/delivery of other implementation strategies. In this paper, our goal was to describe our developmental process, key content principles for consumer engagement in implementation, and how what we learned in each stage informed key design decisions for final tools.

## 2. Developmental process overview

### 2.1. Guiding framework

**Figure 1:**
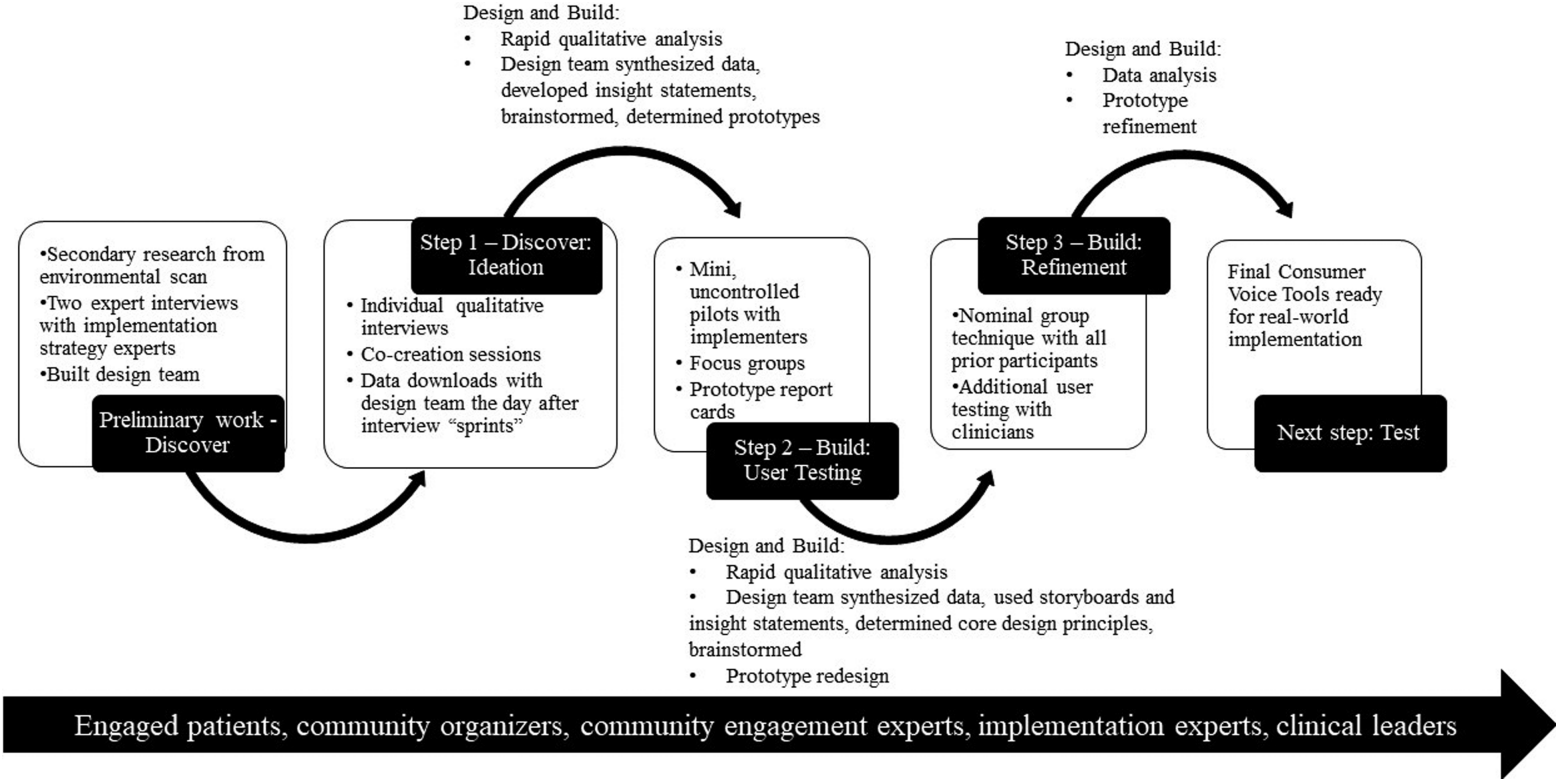
Sequential steps using research methods and human-centered design to develop consumer voice.

We developed Consumer Voice from April 2021 to November 2022 using a multistep, iterative process combining health services research and human-centered design methods. Our process was consistent with the Discover, Design, Build, and Test human-centered design framework for implementation (20). This framework suggests four phases in developing solutions to implementation problems, each with a different focus. The first is to discover targets for change or of need by identifying needs and perspectives of people involved and the context for implementation. The second and third phases are focused on design—synthesizing information learned in the Discover phase and then coming up with ideas and principles for potential solutions—and then building prototypes of solutions. Activities can cycle back and forth between Design and Build phases new data gathered through user testing is used to modify or redesign solutions. The final Test phase involves evaluating high-fidelity prototypes in a real-world implementation context. In this paper, we describe activities in the Design, Discover, and Build phases of Consumer Voice—see Figure 1, incorporating co-creation with potential end-users with limited tools in constrained time settings (akin to alpha testing) as well as a step in which actual end-users interacted with the tools in their own environment (akin to beta testing).

### 2.2. Research and design team roles in decision making and prototyping

We had a research team and a design team that served distinct functions and contained overlapping members. The research team designed and executed data collection and analysis using traditional health services research methodologies in each step. The design team then synthesized research data in the context of their lived or professional experience related to the topic, generated key insights from data related to Consumer Voice development, and brainstormed and made decisions about prototype solutions to address those topic areas. The research team made all prototypes and refinements. The research team consisted of the principal investigator of this study, a doctoral-level clinical psychologist (ENW), a research assistant with a bachelor's degree in sociology (IAB), and a qualitative methodologist trained in implementation science (KLD). The design team consisted of the research team plus another clinical psychologist and implementation researcher (RSS); one consumer as a co-design participant—a military veteran consultant who was a former seaman in the U.S. Navy as well as engaged in women veterans outreach and certified in health benefits administration (CS); a second consumer as a co-design participant—a military veteran consultant with a juris doctorate who was a retired colonel in the U.S. Army and National Guard (DC); a psychiatrist and implementation researcher (JEK); and an anthropologist who engages community members in implementation science (CW).

### 2.3. Participants and recruitment

We engaged participants with diverse experiences throughout our multistep development process. Participants included members of the target end user group, which included those with knowledge of and/or need for safety planning to prevent suicide in rural Arkansas, as well as individuals with knowledge that would generalize to consumer engagement or implementation in any setting. Specifically, we recruited: (1) veterans living in rural Arkansas who experienced suicidal thoughts or attempts; (2) Arkansas community members involved in suicide prevention (e.g., state Veterans Service Officers, community organizers who were also veterans); (3) mental health leadership at VHA rural clinics; (4) suicide prevention providers and champions at the main central Arkansas VHA medical center, and at the national level; (5) implementers who would theoretically use Consumer Voice in their work; and (6) community engagement experts in any area.

Using a respondent-driven, non-probabilistic approach, we reached out to relevant professional groups, community organizations, or established veteran contacts in the community via email or social media, asking them to suggest potential participants. After generating a list of people who might meet criteria, the research team made phone calls to screen for eligibility. We screened for eligibility using simple questions consistent with the inclusion criteria shown in Table 1 (e.g., For implementers, “Do you have practical experience implementing new treatments/programs into practice and considered or attempted to engage consumer groups in this process?”). For veterans with lived experience, we used multiple questions about (1) their military status, (2) their zip code, and (3) whether they ever thought about ending their life, planned to end their life, or attempted suicide before. We compared their zip code to the Rural Urban Community Area (RUCA) code database to determine if their residence was considered rural (21). Veterans were eligible for our study if they lived in an area with a RUCA code 4–10, indicating large, small, and isolated rural towns. Veterans were not eligible for the study if they appeared to have trouble remembering key parts of the screening conversation or demonstrated memory impairments that could be due to cognitive or substance use issues, as we believed this compromised their ability to give informed consent. We were also prepared to exclude Veterans who were high risk for suicide using a suicide risk protocol, although no one met this criterion.

**Table 1:** Participant groups’ inclusion and exclusion criteria and sample size for steps 1, 2, and 3.

Participant group	Inclusion criteria	Exclusion criteria	Step 1	Step 2^a^	Step 3^b^
Veterans with lived experience and community members	Veterans living in rural Arkansas who experienced suicidal thoughts or behavior or their caregivers, family, or peers; Arkansas community members involved in assisting with preventing Veteran suicides (e.g., clergy, state Veteran Service Officers)	Acutely high risk for suicide at the time of study activities; cognitive impairment or substance use that impedes study activities	*N* = 10	n/a	*N* = 8
Implementers and implementation experts	Persons with research or practical experience implementing new treatments/programs into practice who have considered or attempted to engage consumer groups in implementation, VHA or non-VHA settings; can reside in any country	Have not considered or attempted to engage consumers in implementation	*N* = 5	*N* = 11^a^	*N* = 1^c^
Community engagement experts	Persons of any discipline trained and experienced in engagement of consumers, communities, and other patient-level stakeholders in research or implementation, VHA or non-VHA settings; can reside in any country	No experience in consumer or community engagement	*N* = 3	n/a	*N* = 2
VHA personnel including suicide prevention champions in rural clinics	Persons employed in VHA, in a national or local role related to suicide prevention or safety planning intervention; included rural clinic mental health leaders	Not employed in VHA, general mental health researcher or employee with no clear expertise in suicide prevention	*N* = 8	n/a	*N* = 3

^a^ These are 11 participants unique from participants in Step 1.

^b^ These participants were from the same sample as recruited in Step 1.

^c^ We sampled much fewer implementers in Step 3 than prior steps because they represented a group with more exposure to consumer engagement in implementation activities and by Step 3, we wanted to sample a group with less exposure to this topic for their “real-world” reactions to Consumer Voice tools.

If participants were eligible, we then assessed if they wanted to participate in the study. If they agreed, they were enrolled. These individuals were recruited for either Step 1 qualitative interviews and co-creation of tools or Step 2 focus groups. Finally, all were recontacted for participation in Step 3 nominal group technique processes for final refinement of tools.

Individuals were engaged in an informed consent process and compensated for each step in which they participated. Consumer and community members were compensated $30 per hour (up to $90 dollars total) and professional implementers and community engagement experts outside VHA were compensated $100 per hour (up to $300). VHA hospital employees were not compensated for research activities when occurring during their official work hours per VHA policy. The study was approved by the Central Arkansas Veterans Healthcare System Institutional Review Board.

## 3. Multistep process and key insights

### 3.1. Preliminary work—discover

#### 3.1.1. Process

We based initial prototypes on themes from preliminary work in an environmental scan from May 2019 to April 2022 on what implementers had already done to engage consumers in implementation efforts in U.S. healthcare systems (6). In the environmental scan, we synthesized data from published literature, publicly available webinars, and surveys or interviews with seven implementers. We also interviewed two implementation strategy experts about how they preferred to learn to consider how best to “teach” consumer engagement strategies to other implementers.

Using human-centered design methods, the design team engaged in a synthesis process by reviewing data from activities, developed key insights from the data, prioritized important concepts, and then collectively brainstormed how to prototype those concepts. Any potential solutions to teach implementers to use consumer engagement implementation strategies were (1) prioritized by the design team and (2) based on “key insights” gleaned from data generated in each step—see key insights from this step below.

#### 3.1.2. Key insights

We generated and prioritized five key insights from our preliminary work. Those five insights were:

1) There are many ways to engage consumers in design/delivery of implementation strategies and not all consumers nor implementers want the most intensive engagement. Environmental scan data showcased a range of intensity of consumer engagement activities, including lower intensity activities such as obtaining unidirectional feedback from consumers about an implementation strategy and higher intensity activities such as using patient and family advisory councils in hospitals, in which patients were voting members on hospital committees where decisions were made about policy or process. Some implementers wanted more intensive engagement, but got feedback that it was neither feasible nor of interest to consumers.
2) There is a recognized need for mentoring and coaching for learning to use consumer engagement implementation strategies. Implementers may not know how to engage consumers in implementation meaningfully. Engaging consumers may be something they have never considered nor been taught. Therefore, it would be important to seek out people who have experience engaging consumers (early adopters) to ask for information on their processes and skillset. Solutions might involve may be of lower intensity (e.g., shadow other experts) or more formal mentorship or longitudinal processes (e.g., ongoing consultation, learning collaborative).
3) Structures and processes to engage consumers need to be empowering for them. Consumers often have the least amount of legitimate power in the implementation process. Implementers usually belong to a health care system or organization that consumers are accessing for their needs to be met. There may be concerns about speaking up on how to improve these systems and how it may impact their care or services. To actively engage in power sharing, it is important to develop a sense of psychological safety, rapport, and activities where consumers are centered and heard, e.g., allowing consumers to select location of meetings, soliciting input from consumers on meetings processes, or co-leading meetings with consumers.
4) It is important to clarify whose voice we are hearing and who they represent. Consider who is the most likely to speak up during the implementation process and who is the most likely to have their feedback listened to and heard. Often the voices most heard during the implementation process are those with more power (e.g., leadership). There may be social characteristics of voices associated with the majority that are most often heard (e.g., cisgender, white, men). It is helpful to check in during the implementation process and ask ourselves and our implementation teams (1) who are we not hearing from? and (2) how can we bring them into the conversation?
5) We are not sure what the best solution is yet to support implementers using consumer engagement strategies. Existing resources to learn about this topic are not synthesized anywhere currently. Another challenge is the innate societal power structures encountered in consumer engagement work. Although we may provide recommendations and ways to consider minimizing power imbalances, those power structures are still in place and implementers often belong to institutions that may have a history of real and perceived harm toward consumers.

With these insights in mind, we agreed to compile prototypes in one location, providing easy and central access to implementers. The design team voted to prioritize and develop two low-tech prototypes that were used as a starting point in Step 1. The prototypes addressed building psychological safety in an implementation team where consumers would be present (insight #3) and building regular check-ins for an implementation team on how they are working together and if consumer voices are being heard (insight #3).

### 3.2. Step 1—discover: ideation (individual interviews and co-creation sessions)

#### 3.2.1. Process

Then, in formal Step 1 of our study, we completed 1-hour interview and co-creation sessions with 26 participants via video conference or telephone from June to September 2021. Inclusion/exclusion criteria and sample size for Step 1 interviews are listed by participant group in Table 1. Sessions were audio recorded, and one interviewer took detailed notes using a template. The purpose of the interview sessions was to refine operational definitions of what tasks might be preferable in “involving consumers or family members in implementation or quality improvement activities,” describe barriers to and facilitators for using these methods, and technical resources needed for Consumer Voice tools. We had two prototypes from our preliminary work we initially showed participants in Step 1, and they suggested new ones as well, so we co-created by either making or refining prototypes with participants during these interview sessions as well (22). We shared video screens in an online platform, consulting participants on what they wanted changed, their response to certain visuals or words, all while making refinements in real-time.

We asked questions about preferred types of consumer engagement and technical or logistic resources needed for consumer engagement in implementation activities. We presented a hypothetical scenario about consumer engagement in implementation activities, asking open-ended questions to inquire into their reactions. We often followed up on responses by probing with “five whys,” posing the question “Why?” five times, thus prompting the participant to share very specific motivations or needs that are not always clear in their initial answer (23). See our supplemental file for interview guide.

Within 4–6 weeks of collecting data from Step 1 interviews, the research team analyzed the qualitative data using a rapid assessment process relying on audio recordings, notes, and summary templates in Microsoft Word software (24). This analytic technique is useful for studies in which there is a time-sensitive demand for creation/modification of a product, yet need for rigor (25). The qualitative analysis team included two coders (ENW and IAB) and a consulting researcher (KLD). The coders were a research assistant who completed a short course in rapid assessment processes for qualitative analysis and a PhD researcher who had taken part in the same short course and had training in other qualitative methodologies (e.g., grounded theory). The consulting researcher was a PhD anthropologist with extensive training and experience using qualitative analysis.

We blended inductive and deductive approaches, first reviewing audio recordings and notes from each interview, importing data into a written template with domains based on our specific interview questions to guide analysis deductively. We integrated new domains as emerging topics were mentioned repeatedly by participants. Before listening to recordings, two coders met to review note summaries and adapted the template as needed, eventually forming a blank master template. For the first five interviews, two coders listened independently to the audio recording, sorting data into the template categories. The coders met and discussed concepts after each interview to develop consensus and agreement and create a final master template for each participant. The coders then divided the remaining interviews between them, each templating all 26 interviews assigned to them independently. One coder reviewed all templates, asking and resolving questions from the other coder, creating a final set of individual templates.

Together, the coders synthesized data from individual templates from each participant across three matrices that addressed different topics: (1) operationalizing consumer engagement in implementation (i.e., who should be involved, how, when, where, and why); (2) suggestions for tools to teach others how to engage consumers in implementation (e.g., online platforms, reading materials, worksheets); and (3) barriers and strengths to anticipate when using the tools, with resources for reference. Within each matrix, coders organized data by participant type to identify patterns within groups (e.g., community members and organizers, implementation experts). The design team met, digested data from each matrix, then synthesized key insights and brainstormed potential solutions to each insight, listed below.

#### 3.2.2. Key insights

We identified five key insights from our Step 1 activities. The insights shared a common theme related to building trusting between consumers and implementers. Specifically, insights included:

1) Implementers need to be prepared to really listen to consumer input and perspectives by responding empathically to consumer concerns. Implementers should resist defending one's practice and/or institution, and instead think about how they would want to be responded to in their own healthcare delivery. Lowering defensiveness would require increasing comfort with consumers seeing the “*dirty laundry*” behind the scenes when implementing innovations. Openly allowing criticism of the practice and/or institution can lead to trust building. Implementers need to use or develop skills to regulate themselves when receiving negative feedback.
2) There are several ways to recruit and engage consumers in implementation efforts. Options include, but are not limited to, using technology to overcome divides among consumers dispersed geographically, setting up feedback loops for local community members to express needs confidentially, providing resources for consumers to attend meetings (e.g., bus passes, tablets), meeting outside traditional work hours and locations, having specific tasks consumers can do in the effort, following through on tasks identified as key by the implementation team, and being thoughtful about how people are arranged to work together to minimize power differentials and increase engagement. Ongoing engagement is one way to build trusting relationships and overcome mistrust.
3) Implementers must work with diverse groups of people involved in the problem to garner different perspectives on the issue and form a more complete understanding of problems and potential solutions. People representing consumers in the implementation process need to be representative of specific populations who are target users of the innovation or evidence-based practice to be implemented. Consumers who had negative experiences with the topic or institution should be included also to fully understand their concerns and glean insights into how to become credible again (e.g., “dissatisfied customers”).
4) Implementers need to showcase how consumer input is valuable to the implementation effort. If there is no discussion or follow through on consumer feedback on how/when/what implementation strategies should be used, it can lead to disengagement and mistrust. Examples of showing how consumer input is valuable included saying explicitly to consumers their voice matters and to please share, moving forward with action based on their input, and providing consumers with feedback of what happened with the input they shared.
5) Implementers must clarify roles of all team members and expectations. Examples included working on formal or informal agreements that communicate clear expectations regarding roles, time commitment, and how work will get accomplished for all involved. This also includes a clear and full orientation for consumers to what work needed to get done, when, how, and why.

Based on the above insights and prototypes voted on by the design team, the research team created new and refined existing prototypes to support implementers. One prototype that was created from our preliminary work was refined further in co-creation sessions with participants, which expanded on practical tips for implementers in creating and assessing for psychological safety among consumers (insight #1). Another prototype provided practical tips for implementers prepare to receive and respond to negative feedback from consumers (insight #1). A third prototype focused on using a visual spectrum to showcase a range of low-to-high intensity engagement strategies, such as one-time brief interactions to long-term equal partnerships (insight #2). A fourth focused on helping implementers balance a greater diversity of consumers involved with a small enough group format to enhance engagement (insight #3). Each insight did not yield a prototype in each step because the design team did not prioritize it above the other insights.

### 3.3. Step 2—build: user testing (with implementers)

#### 3.3.1. Process

In Step 2, we asked implementers to pilot the prototype of Consumer Voice tools briefly in their own work and share feedback through focus groups. We recruited 11 participants to use full prototypes of Consumer Voice tools; focus groups were hosted May–June 2022. Using experience sampling (26), participants comprised implementers who would theoretically use Consumer Voice in their work inside VHA or outside VHA settings. Participants were given 2–4 weeks to use the Consumer Voice tool prototypes developed in Step 1 however they wanted. Although not required, we also asked participants to take notes specifically on the following questions as they used the tools: “Can you use this in your job?” and “What is missing?” Four participants provided written feedback.

Across focus groups, we asked participants the same three questions: “Could you actually engage consumers in your planning using these tools, and why or why not?” “What about the format needs to change and how?” “Did you feel confident about selecting modules, and why or why not?” Participants responded verbally. We used a qualitative rapid assessment process similar to what we used in Step 1, although with the goal to capture all feedback in a comprehensive manner rather than identify repeating ideas. Coders used note summaries and audio recordings from focus groups to populate one master template for all focus group qualitative and written data. The template captured: things they liked, things that were missing or needed changing, formatting, and other tools we might create. We summarized user feedback in an 11-page, single-spaced document including feedback on aspects they liked, things that needed improvement, and minor wording or formatting changes. The design team synthesized the data and generated additional insights and brainstormed prototype changes. Ultimately, we made every change the user testers suggested prior to showing the revised tools to users again in Step 3 rather than only prioritizing key insights.

#### 3.3.2. Key insights

Users in Step 2 liked that the tools that were communicated via slide sets and word documents. They felt that tool content was almost comprehensive, and they perceived the value of the tools to help with meaningful engagement with consumers. As one participant said, “*If someone uses the materials, it's going to protect [consumers] from being invited to be a part of this in a tokenistic way*.” Examples of aspects users did not like included being unsure how to start, as they felt the materials were overwhelming at first. They also felt there was not enough detail on assessing for power differentials between consumers and implementers. They identified key content that was absent from existing tools, such as information on how to compensate consumers for their contributions to the implementation process. By this step, we also had enough data from multiple perspectives to clarify our “design principles”—core elements that our solutions should follow in terms of how they presented materially. Our design principles were as follows:

• Materials must be “bite-size”—just enough to learn something, then have depth and examples for people who want to dig deeper.
• Simpler is better regarding web functionality and wording.
• Do not be prescriptive—give options for how to work with consumers.
• Use examples to showcase application of concepts.

Based on user feedback about content, we added new prototypes and refined existing ones. One new prototype was written guidance and a templated worksheet for implementers to consider and decide how to compensate consumers who help design or deliver implementation strategies. Another prototype was an entirely new module entitled “How to Use Consumer Voice” to address the concern that materials were overwhelming and needed more orientation. Based on user feedback about design principles, we added a real-world example to every module showcasing how to apply a concept, and ensured new prototypes adhered to the above design principles.

### 3.4. Step 3—build: refinement (focus groups using nominal group technique)

#### 3.4.1. Process

In Step 3, we attempted to reconvene all participants from prior Steps 1 and 2 to share updated Consumer Voice tools and conduct a nominal group technique process to vote on the most feasible and important components of final prototypes (21, 22). Participants from all prior steps were invited to independently review revised tools and participate in a 1-hour group feedback session November–January 2023. See Table 2 for participant demographics. To reduce power differentials and dual relationships with each other, feedback sessions were conducted separately for professionals and for consumers or community members. To increase participant inclusion and generate feedback from every participant, we used a nominal group technique process. Nominal group technique included the following steps. First, we asked one open-ended exploratory question: “What are the areas we need to improve upon in the Consumer Voice tools?” Participants had 5–10 min of quiet time to independently generate ideas. Second, participants reported their ideas orally to the larger group without discussion. Third, the group facilitator invited participants to ask questions to better understand an idea another participant had shared or elaborate upon their own comment. Finally, each person voted publicly on their top ideas to prioritize for impact. In the final step, we also collected demographic information from participants. We audio recorded feedback and took written notes. We ended each session with a list of prioritized recommendations.

**Table 2:** Demographic characteristics of 14 participants who completed steps 1 and 3^a^.

Demographic characteristic	*N* (%)
Age	*Mean* = 41 years, *Range* = 34–51 years
Military veteran status	
Enlisted, non-commissioned officer, discharged	3 (37.5%)
Retired	1 (12.5%)
Part of professional organization serving veterans	2 (25%)
Family member or friend of veteran	1 (12.5%)
Other	2 (25%)
Gender identity	
Man	3 (38%)
Woman	5 (63%)
Disability status (mental, physical, cognitive)	
Yes	4 (50%)
No	3 (37.5%)
Did not report	1 (12.5%)
Racial identity	
Asian	1 (12.5%)
American Indian or Alaska Native	2 (25%)
White	6 (75%)
Sexual identity	
Straight or heterosexual	6 (75%)
Bisexual	1 (12.5%)
Lesbian, gay, or queer	1 (12.5%)
Geographic location	
Rural	2 (25%)
Urban	5 (62.5%)
Did not report	1 (12.5%)

^a^ We did not collect demographic data from participants in Step 2 who were all implementers or implementation experts.

Coders compared recommendations across all groups and created a matrix comparing recommendations between consumer/community members and professionals. The goal was to capture a subset of priority feedback areas rather than to comprehensively capture all feedback. Quantitative analysis was used to count final votes and to sum the frequency and percentages of votes.

#### 3.4.2. Key insights

Our nominal group process yielded a prioritized set of 8 refinements for the tools, four of which focused on content and four focused on usability. Content refinements included:

1. Emphasize which content helps build a trusting relationship with consumers and include more content on assessing power imbalances.
2. Reframe sections on “leading meetings” to clarify that people who share negative feedback are not “obstructive” but offer critical feedback based on legitimate lived experiences.
3. Emphasize content conveying that there are multiple avenues to engage consumers and avenues used should be sensitive to consumers’ time limits, literacy, physical ability, etc.
4. Create a brief exercise for people to share with each other their own histories of engagement or work within their organization to help understand motivations and skills early.

Refinements focused on improving usability of the tools through better design included:

1. Condense content without removing any substantive details—one idea was to use audio voiceovers for slides.
2. Provide additional guidance to orient the user and help them know where to start.
3. Incorporate more examples from settings other than healthcare.
4. Make titles of modules more specific and action oriented.

**Figure 2:**
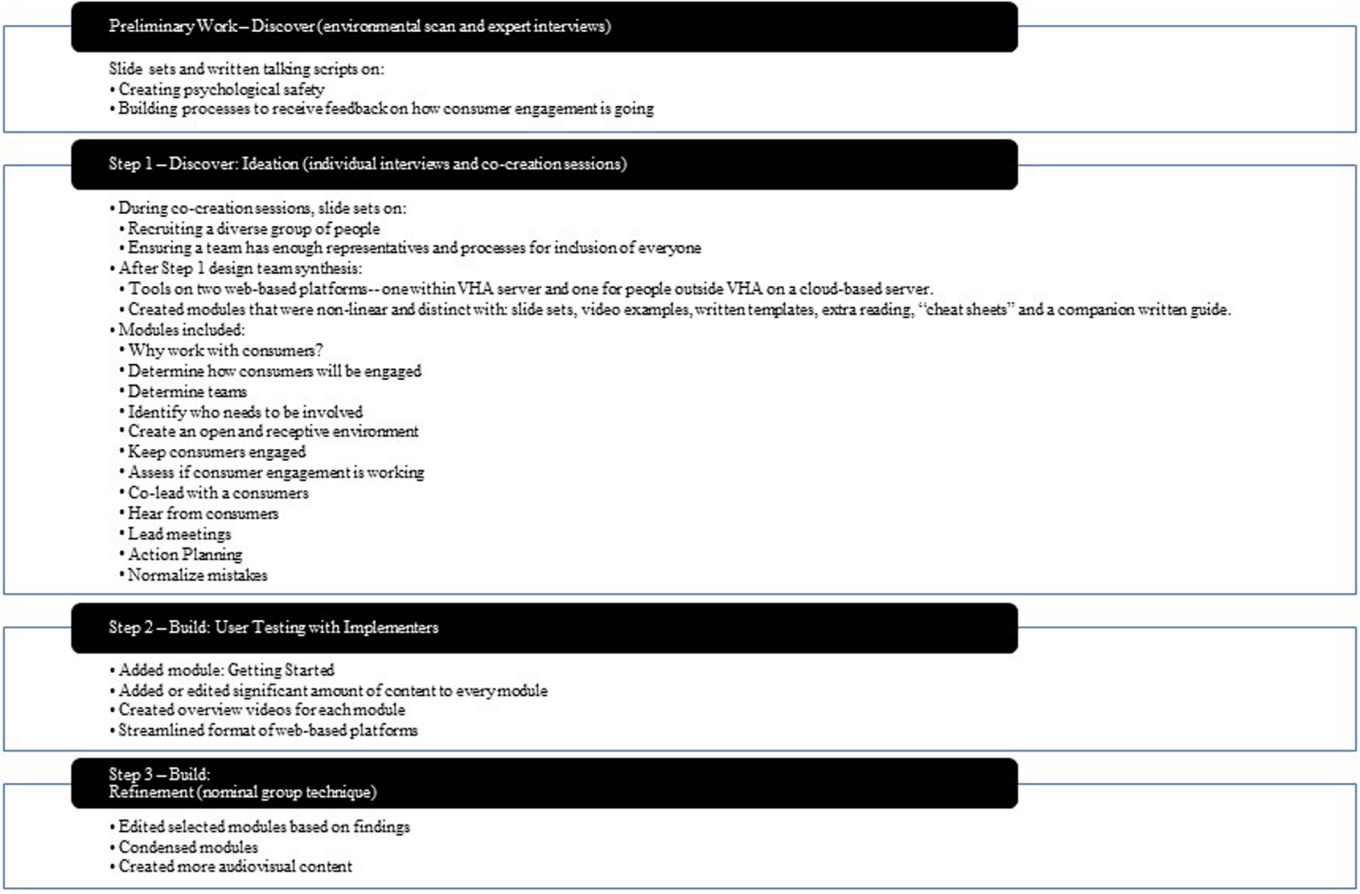
Prototype evolution step-by-step.

We incorporated those suggested improvements into a final version of Consumer Voice tools, which are currently freely available to users outside VHA online (27) and users within VHA on a Sharepoint website (28). See Figure 2 for evolution of key insights and prototypes over research and design activities.

## 4. Discussion

We blended human-centered design approaches with health service research methods to design a compendium of tools, Consumer Voice, to support implementers of new innovations in how to “involve consumers or family members in implementation or quality improvement activities” in a centralized location. Through discovery from multiple data sources and repetitive cycles of designing and building prototypes, we built the same, free compendium of tools on two different online platforms—one within VHA server (28) and one for people outside VHA on a cloud-based server (27). We identified principles for designing solutions (e.g., how the tools should function and what they should be like) and essential content (e.g., there are multiple ways to recruit consumers into implementation efforts, find a diverse set of consumers representative of the population you are trying to serve). The tools are modular and nonlinear tools allowing for self-guided learning tailored to beginner, intermediate, or advanced experience with consumer engagement.

Consumer Voice tools offer great specificity on the “what to consider” and “how to” for the consumer engagement implementation strategy “involving consumers or family members in implementation or quality improvement activities” (4). In other words, Consumer Voice offers multiple suggestions for implementers to engage consumers in the design/delivery of implementation strategies, which might include other consumer engagement implementation strategies (e.g., using mass media) or system-facing implementation strategies (e.g., redesign workflow, shadow other experts). It would be helpful for implementers using Consumer Voice to track strategies that emerged from their use of the tools.

Consistent with the Discover, Design, Build, and Test framework for human-centered design in implementation efforts, we will continue this work with a formal test of Consumer Voice. At the time of this publication, we are conducting a feasibility and acceptability assessment of Consumer Voice tools in the context of improving reach and quality of safety planning intervention among rural Veterans at moderate risk for suicide in VHA (29). We will combine Consumer Voice tools with Implementation Facilitation to address all levels of the implementation context. Another next step for research on consumer engagement in implementation, whether using Consumer Voice or other tools, is to assess their impact on implementation and effectiveness outcomes. The need for data on consumer-level outcomes of involving consumers in the design/delivery of implementation strategies was noted in a systematic review on this topic, which found that outcomes were typically reported for clinic/hospital/system of care but not for patients’ experiences, behaviors, or health (30).

One interesting finding was that almost all key insights generated by the design team from the environmental scan and individual interviews with co-creation sessions explicitly served to build trust between consumers and professionals representing institutions. This is especially noteworthy if implementation activities are to reduce healthcare disparities and improve health equity through enhanced trust for consumers who have experienced significant neglect or harm from institutions providing services (e.g., 31–33). Key insights that ultimately informed Consumer Voice tools align well with a recent proposed theory of change related to trust-building in implementation efforts (34). Authors of this theory propose that to build trust during implementation efforts, implementers must focus on *what* (technical strategies) they do to engage with others and *how* (relational strategies). Specifically, technical trust-building strategies involve frequent interactions, responsiveness, demonstrating expertise, and achievement of quick wins; while relational strategies involve showcasing vulnerability, authenticity, bi-directional communication, co-learning, and empathy-driven exchanges. Many of our prototyped tools suggest these very technical strategies (e.g., make community agreements, discuss tactics to keep engagement confidential, compensate consumers) and ways to embody the relational strategies (e.g., balancing group discussion with options for anonymous or individual feedback, emotionally regulating oneself before a meeting when asking for critical feedback). An interesting next step in research would be to assess the impact of consumer engagement in implementation activities on trust, specifically, or assess trust as a moderator of change in other consumer-level or organizational-level outcomes.

Some design principles favored efficiency and clarity, which were not surprising given busy settings where people work. One design principle—do not be prescriptive about how consumers should be engaged, but instead, give options—is consistent with documented examples of consumer engagement in implementation efforts. There is a range of intensity of implementation activities, and the most intensive consumer engagement implementation strategy is not always feasible or ideal to either consumers or implementers, given the context (35). In Bombard et al.'s (2018) systematic review, intensity of engagement appeared to influence outcomes of the quality improvement or implementation effort. Discrete products such as brochures or policy documents typically derived from low-level (consultative) engagement, whereas care process or structural outcomes such as enhanced care or shared governance typically occurred when there was high-level (co-design) engagement (30). Lower intensity consumer engagement (in research, not implementation), such as consultation with unidirectional feedback, have been considered by other scholars to represent even non-participation or something symbolizing participation by consumers without meaningful contribution (36). Our results supported this conclusion and yet, also, recognized there is variability across implementers *and* consumers regarding their ability and interest in higher intensity consumer engagement implementation strategies.

### 4.1. Limitations

This study has limitations. For one insight garnered in Step 1, “There is a recognized need for mentoring and coaching of learning,” we did not design any solutions yet, as we believed the other insights could be addressed initially through a compendium of tools and we did not have the person or financial capacity to develop an ongoing mentoring or coaching program. Our qualitative analysis used rapid extraction and templates, rather than using written transcripts or deeper coding, so it is possible we missed some more nuanced viewpoint from participants. And yet, the rapid assessment process was generally well-suited as a data extraction method (vs. true qualitative coding and thematic analysis) because the questions and data generated from interviews were more straightforward feedback about the topic. Also, the design process took place in the U.S. state of Arkansas and focused on strategies to engage consumers in safety planning to prevent veteran suicide. Although we believe our process is applicable to other patient populations and settings, we also cannot speak to how the product generated through our human-centered design approach would compare to products generated using other strategies. We also collected demographics from participants in the final Step 3, resulting in missing demographic descriptors of some participants who contributed to Steps 1 and 2. Future testing of the effectiveness of Consumer Voice in multiple settings and with larger samples of implementers and consumers is needed prior to widespread adoption.

### 4.2. Conclusions

Including consumers in design/delivery of implementation strategies is increasingly recognized as essential for achieving equitable implementation and effects of innovations. Yet, there is still a great omission of principles and practical tips to engage consumers in implementation activities, which is essential if consumer engagement implementation strategies are going to have their desired effects. This study fills this gap by using a “consumer focused” approach to develop much-needed guidance for implementers to use as they begin to include consumers engagement implementation strategies, informed through meaningful consumer input, in their future implementation efforts. Although the resulting product, Consumer Voice, was developed in the VHA healthcare context and specifically focused on including rural Veteran patients in improving implementation of a suicide prevention intervention, our process included participants outside VHA and mental health care settings to increase applicability to other settings or health topics.

## Data Availability

The original contributions presented in the study are included in the article/**Supplementary Material**, further inquiries can be directed to the corresponding author.
