# Supplementary material for: Increasing consumer engagement: tools to engage service users in quality improvement or implementation efforts
Source: Front Health Serv. 2023 Jul 25;3:1124290. doi: 10.3389/frhs.2023.1124290 (PMC10407803; doi:10.3389/frhs.2023.1124290)
Supplement: Supplementary file 1 [file Datasheet1.docx]

**Supplemental File 1: Qualitative Interview Guide for Step 1 Interviews**

**Version: Patients/Veterans/Caregivers/Community Members**

This interview guide is for stakeholder interviews for Step 1 to create a draft of Consumer Voice for Safety Planning Intervention (SPI) implementation. Interviews will be semi-structured, allowing the interviewer to follow new areas of inquiry as they emerge naturally. The interview guide will be modified over time as we complete interviews as one way to enhance reliability of results. Although there is an order to the interview guide, in practice, the interviews will flow more naturally, and the interviewer will refer to the guide to make sure that all the topics are introduced and covered in the interview.

Note: The same content was asked about in each interview. Questions varied by who participant was – e.g., if a patient in the healthcare system, the questions were worded so they reflected the patient care role and if an implementer, questions were worded to reflect an implementer’s role.

1. Preferred Types of Consumer Engagement

*I will start broad, and as I ask questions, I might sometimes ask you “why” a lot, just to really understand where you are coming from.*

*Imagine someone from VA mental health service calls you and says, “We’ve got this new therapy to help patients who come to VA with suicidal thoughts. We’d like to have your voice and input as we design how it gets rolled out.” In your dream scenario, how would you like this to go?*

*Why? Why? Why? Why? Why?*

*What* *activities would you like to be involved in when VA is designing how they will implement a new treatment?*

*Are there any kinds of involvement you would be opposed to?*

*Have you ever been involved before in something like this, and if so, what did you think of it?*

*Probe: What would be a good way to organize different people with different roles?*

*If you were to be involved in this, how would you want someone from VA reach out to you about this? Probes: Would you want to be on a contact list, as needed? Are there any steps in the implementation process where you think patient involvement would be especially difficult? Why are you concerned about this?*

1. Technical Resources for Consumer Voice

*We’ve thought of ideas to get patients and families and community members involved in implementation and see what you think. I would like to know your thoughts about some of them. What are your thoughts about:*

- *Attending a 2-hour meeting where we’d have group discussion and structured exercises, like voting and writing things down in small groups, to figure out the best strategy? Good, Bad, or Not Sure?*
- *When would you like these meetings to be? Where? With whom? How many people is too many? Good, Bad, or Not Sure?*
  - *Patients only vs mixed stakeholder*
  - *Ongoing vs one-time*
- *To explain the way a new treatment might get implemented, would you prefer a video, for it to be written down, or for someone to talk about it verbally with you?*
